# Transcriptomic Analysis of Extracellular RNA Governed by the Endocytic Adaptor Protein Cin1 of *Cryptococcus deneoformans*

**DOI:** 10.3389/fcimb.2020.00256

**Published:** 2020-06-23

**Authors:** Muxing Liu, Zhengguang Zhang, Chen Ding, Tuo Wang, Ben Kelly, Ping Wang

**Affiliations:** ^1^Department of Plant Pathology, College of Plant Protection, Nanjing Agricultural University, Nanjing, China; ^2^College of Life and Health Sciences, Northeastern University, Liaoning, China; ^3^Department of Chemistry, Louisiana State University, Baton Rouge, LA, United States; ^4^Department of Microbiology, Immunology, and Parasitology, Louisiana State University Health Sciences Center, New Orleans, LA, United States; ^5^Department of Pediatrics, Louisiana State University Health Sciences Center, New Orleans, LA, United States

**Keywords:** extracellular RNA, endocytic protein, fungal intersectin, intracellular trafficking, fungal pathogenesis

## Abstract

Membrane vesicles are considered virulence cargoes as they carry capsular and melanin components whose secretory transport is critical for the virulence of the human fungal pathogen *Cryptococcus* species. However, other components of the vesicles and their function in the growth and virulence of the fungus remain unclear. We have previously found that the cryptococcal intersectin protein Cin1 governs a unique Cin1-Wsp1-Cdc42 endocytic pathway required for intracellular transport and virulence. Using RNA sequencing, we compared the profiles of extracellular RNA (exRNA), including microRNA (miRNA), small interference RNA (siRNA), long noncoding RNA (lncRNA), and messenger RNA (mRNA) between the wild-type (WT), and derived Δ*cin1* mutant strains of *Cryptococcus deneoformans*. Seven hundred twelve miRNAs and 88 siRNAs were identified from WT, whereas 799 miRNAs and 66 siRNAs were found in Δ*cin1*. Also, 572 lncRNAs and 7,721 mRNAs were identified from WT and 584 lncRNAs and 7,703 mRNAs from Δ*cin1*. Differential expression analysis revealed that the disruption of *CIN1* results in many important cellular changes, including those in exRNA expression, transport, and function. First, for miRNA target genes, Gene Ontology (GO) and Kyoto Encyclopedia of Genes and Genomes (KEGG) pathway enrichment analysis revealed that cellular processes, components, and macromolecular functions are the most affected pathways. A higher number of genes were involved in the intracellular transport of endocytosis. Second, the results of GO term and KEGG analysis of differentially expressed lncRNA target genes and mRNA genes were consistent with those of miRNA targets. In particular, protein export is the topmost affected pathway among lncRNA target genes and one of the affected pathways among mRNA genes. The result of quantitative real-time reverse transcription PCR (qRT-PCR) from 12 mRNAs tested is largely agreeable with that of RNA-Seq. Taken together, our studies provide a comprehensive reference that *Cryptococcus* secretes abundant RNAs and that Cin1 plays a critical role in regulating their secretion. Given the growing clinical importance of exRNAs, our studies illuminate the significance of exploring this cutting-edge technology in studies of cryptococcal pathogenesis for the discovery of novel therapeutic strategies.

## Introduction

*Cryptococcus* spp. are encapsulated basidiomycetous fungi that infect both the healthy people and immunocompromised individuals, causing meningoencephalitis (Perfect, 1989). Virulence of *Cryptococcus* is multifaceted, with the production of the polysaccharide capsule, melanin pigment, and extracellular proteinases characterized as the common virulence factors (Kozel, 1995; Buchanan and Murphy, 1998; Lengeler et al., 2000). The elaboration of these virulence factors depends on intact intracellular transport, including exocytosis and endocytosis, which is a highly conserved and essential cellular process. Endocytosis is a process in which living cells uptake foreign materials through the invagination of the plasma membrane (PM) to form vesicles, whereas exocytosis is the release of vesicle contents to the cell exterior through vesicle fusion with PM (Oka and Krieger, 2005). Normal intracellular transport is required for the prolific cellular growth and differentiation, as well as for the pathogenicity of infectious microbes. For example, the uptake of transferrin provides iron necessary for the parasitism of *Plasmodium falciparum*, a unicellular protozoan parasite (Rodriguez and Jungery, 1986). The secretory transport of glucuronoxylomannan (GXM) and aspartic proteinases (SAPs) is considered to be important for capsule formation, a virulence factor of *Cryptococcus* spp. and the virulence of *Candida albicans*, respectively (Schaller et al., 2005).

Intracellular transport is also highly organized and complex involving the concordant function of many protein partners, particularly, the endocytic adaptor proteins, including human intersectin 1 (ITSN1), and cryptococcal Cin1 [reviewed in Wang and Shen (2011)]. These proteins contain multiple domains that couple endocytic uptake with secretion, interconnect transport with actin cytoskeleton regulation, and interact with signaling events mediated by Rho/Rac/Cdc42 family GTPases (Bourne et al., 1990; Jenna et al., 2002; Huang and Cai, 2007; Kaksonen, 2008). Cryptococcal Cin1 is a multi-domain adaptor protein that plays a pleiotropic function in the growth, transport, and production of virulence factors of the fungus (Shen et al., 2010). Previous studies also demonstrated that Cin1 functions upstream of Wsp1, a homolog of human GTPase-binding domain (GBD)-containing Wiskott–Aldrich syndrome protein (WASp), and Cdc42 to regulate actin polymerization, and dynamics (Shen et al., 2011, 2012). However, other functions of Cin1 remain unknown.

Extracellular vesicles (EVs), including microvesicles and exosomes, are spheroid lipid membrane structures containing cytoplasmic and membrane proteins, phospholipids, metabolites, and nucleic acids. Microvesicles (50–1,000 nm in diameter) are formed by the outward budding of the surface PM, whereas the smaller exosomes (40–120 nm in diameter) are intraluminal vesicles primarily formed by the fusion of the multivesicular membrane or the outward budding of the surface PM [reviewed in Ibrahim and Marban (2016), Quesenberry et al. (2015)]. Recent studies also characterized apoptotic bodies (1–5 μm in diameter), the by-products of cell disassembly during apoptosis, as EVs (Poon et al., 2014). EVs and vesicular RNAs are recognized to be important in diseases, including cancer, neurodegenerative disorders, and infectious diseases, as they carry signals that not only identify themselves but also are capable of altering the function of targeted cells (El Andaloussi et al., 2013; Barile and Vassalli, 2017). Previous studies identified at least 1,244 and ~2,000 vesicular extracellular RNAs (exRNAs) from *Cryptococcus deneoformans* and *C. albicans*, respectively (Jiang et al., 2012; Peres da Silva et al., 2015). We have also identified ~3.3 million small exRNAs from two clinical strains of *Rhizopus delemar* in a previous study (Liu et al., 2018).

To further characterize Cin1 function in secretory transport relevant to the virulence of *Cryptococcus*, we performed next-generation sequencing of exRNAs from *C. deneoformans* wild-type (WT) and Δ*cin1* mutant strains. We showed that *Cryptococcus* secretes abundant RNAs, including microRNA (miRNA), small interference RNAs (siRNA), long noncoding RNA (lncRNA), and messenger RNA (mRNA) and that Cin1 plays a regulatory role including that in secretion. Based on the emerging importance of exRNA as a determinant of various biological processes, including pathogenesis, further exploration of differential exRNA expression and target characterization are highly warranted for *Cryptococcus* species.

## Results

### Small Extracellular RNA Characterization, MicroRNA Identification, and Target Prediction

EVs are heterogeneous nanoparticles naturally released from cells during growth, and they carry cargoes containing nucleic acids, proteins, lipids, and other metabolites. EVs were isolated from *Cryptococcus deneoformans* WT (JEC21) (Kwon-Chung et al., 1992) and the Δ*cin1* strains (Shen et al., 2010) grown in liquid yeast peptone dextrose (YPD) for 3 days at 30°C with shaking (225 rpm), and RNA extraction and size fractionation were all similar to those described previously (Liu et al., 2018). RNA quality assessment, cDNA synthesis, library construction, and RNA sequencing were performed by the Beijing Genome Institute (BGI, Shenzhen, China).

**Figure 1:**
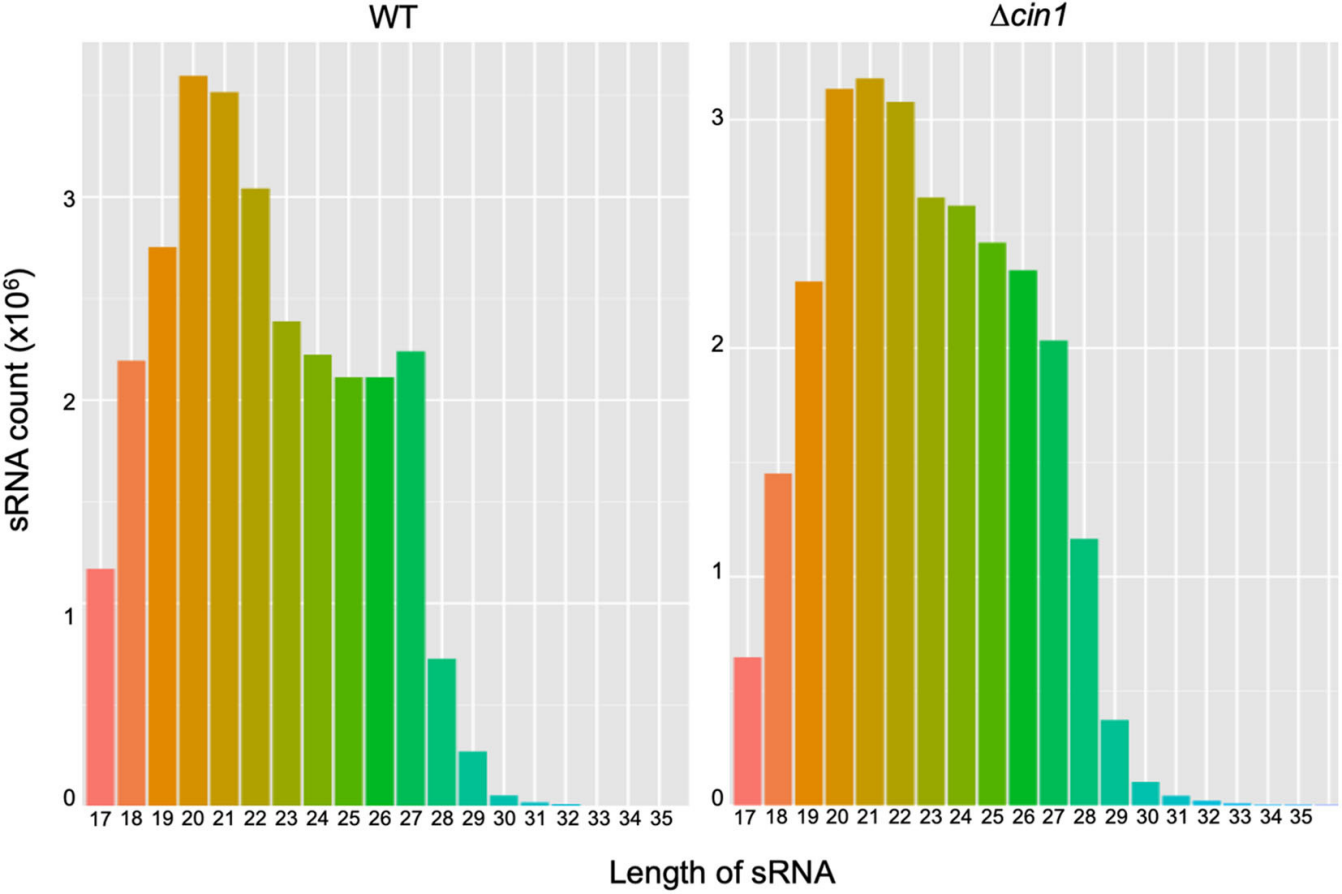
A comparison of extracellular small RNA (sRNA) length distribution between the *Cryptococcus deneoformans* WT (JEC21) and Δ*cin1* mutant strains. The majority of sRNA is within the range of 19- and 22-nt in length, with 20 nt as the major size group in JEC21 (left panel) and 21 nt in *cin1* (right panel). The *x*-axis indicates tag lengths, and the *y*-axis indicates tag read numbers.

A BGISEQ500 platform was used for RNA-Seq of secretory small RNA (sRNA). Approximately 27.4 and 27.1 million sRNA clean reads were obtained from WT and Δ*cin1*, respectively. The clean reads accounted for ~93–94% of the raw reads, with ~96% mapped to the genome of *C. deneoformans*, suggesting a reasonable sequencing depth and accuracy. The median lengths of sRNAs were 20 nucleotides (nt) in WT but were shifted to 21 nt in Δ*cin1*, with a minimum length of 17 nt and a maximum length of 30 nt (Figure 1). Approximately 90–92% of sRNAs were mapped to intergenic regions, whereas 7.5–9.4% were mapped to exons and 0.5–0.6% to introns.

**Figure 2:**
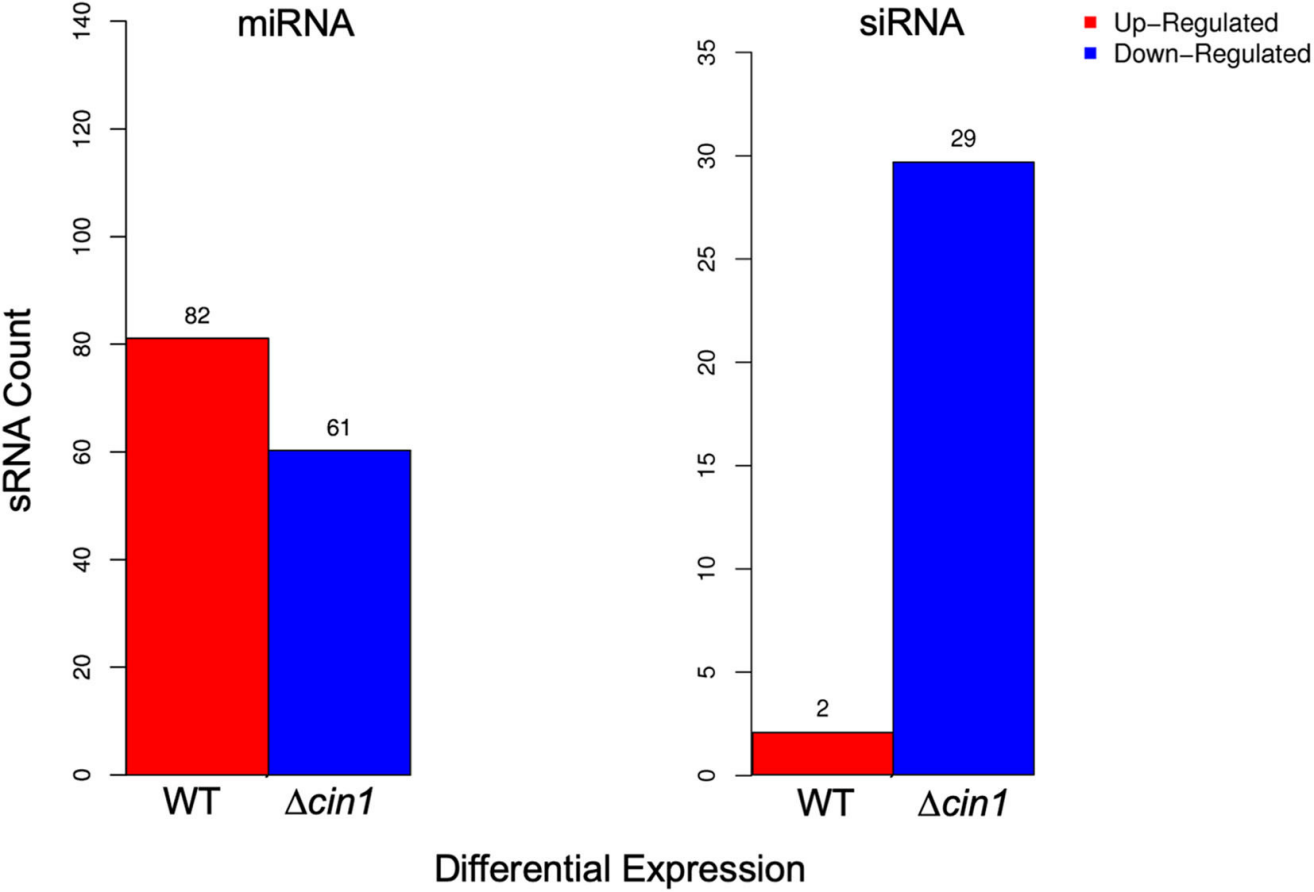
Differential expression of extracellular small RNA (sRNA). Differentially expressed microRNA (miRNA) (left graph) and small interference RNA (siRNA) (right graph) between the wild-type (WT) and Δ*cin1* strains. Red color represents upregulation, and blue color represents downregulation. The expression level is calculated using TPM (transcripts per kilobase million; 't Hoen et al., 2008).

The clean reads were mapped to the sRNA reference database to identify 690 and 787 known miRNAs from WT and Δ*cin1*, respectively (Tables S3, S4). Both strains also contain 12 previously unknown novel miRNAs (Table S5). In addition to miRNAs, 100 siRNAs were identified (88 from WT and 66 from Δ*cin1*, respectively) (Table S6). None of the siRNAs were previously described. Similar to miRNAs, siRNAs are also short duplex RNA molecules that exert gene silencing effect at the post-transcriptional level by targeting mRNA. However, the major difference between siRNAs and miRNAs is that the former are highly specific with only one mRNA target, whereas the latter have multiple targets (Lam et al., 2015). Differential expression screening revealed that WT has more upregulated miRNAs (by 82) than Δ*cin1* but less downregulated (by 61) than Δ*cin1* (Figure 2, left graph). The opposite was true for siRNA: more downregulated (by 29) in Δ*cin1* than WT (Figure 2, right graph). The functional significance of such differential expression is not yet known; however, studies in mammalian systems suggested that miRNA is required for tissue homeostasis as the expression levels of many tissue-restricted miRNAs are usually downregulated owing to illness (Hammond, 2015).

### Functional Annotation of Extracellular MicroRNA Targets

**Figure 3:**
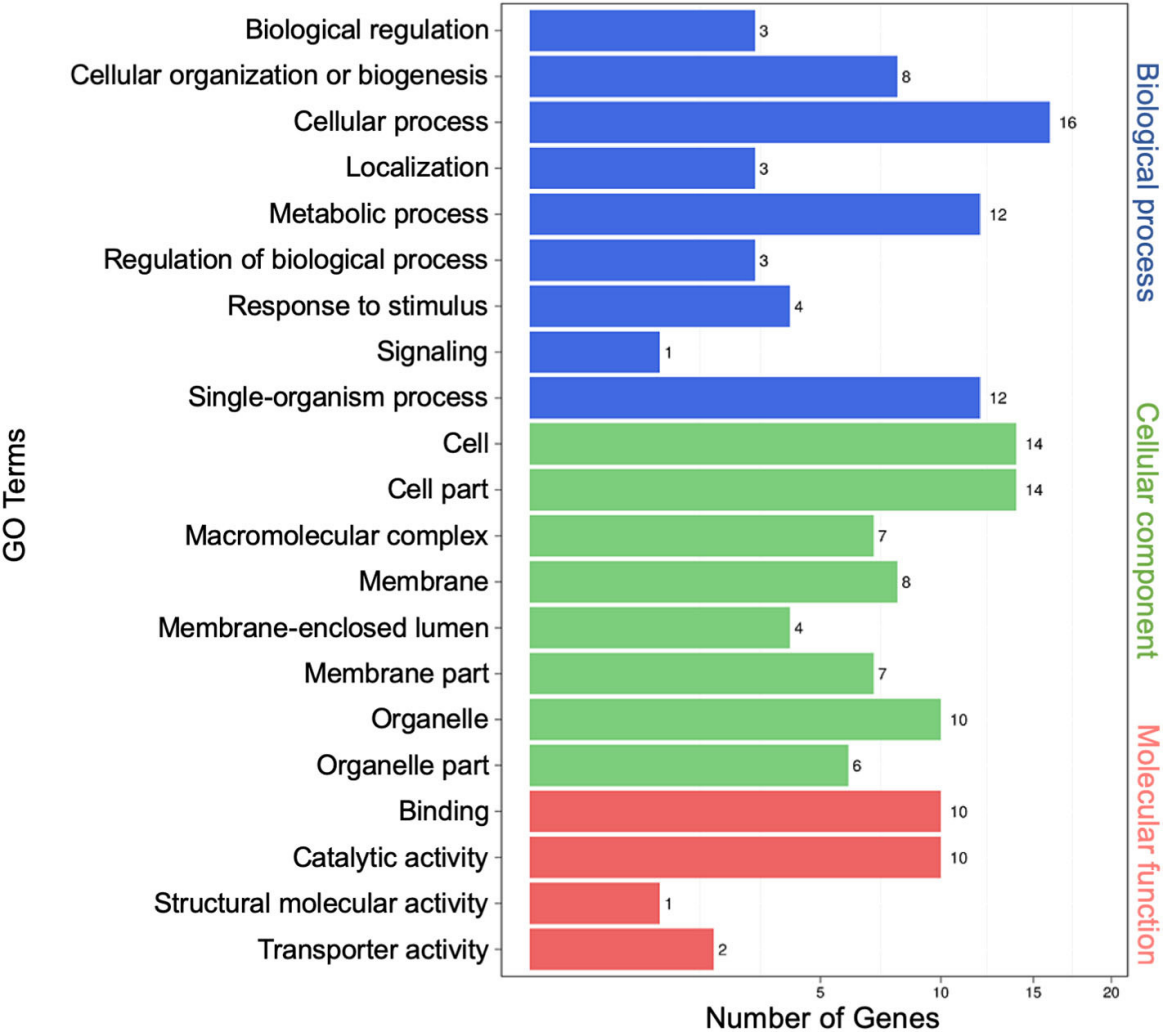
Gene Ontology (GO) analysis of differentially expressed extracellular microRNA (miRNA) target genes. The left vertical coordinates refer to GO terms, the bottom horizontal coordinates are the numbers of differentially expressed genes, and the right vertical coordinates indicate the class of GO terms.

To characterize possible functions of secretory miRNAs, TargetFinder (Fahlgren and Carrington, 2010; Kielbasa et al., 2010) was used to identify ~5,732 miRNA targets out of ~10, 527 known miRNA counts. To categorize the putative functions of miRNA targets, Gene Ontology (GO) term enrichment analysis was performed. In all, 21 GO functional categories belonging to three main categories (biological process, cellular component, and molecular function) were identified. For biological process, the top three functional categories were “cellular process” (16), “single-organism process” (12), and “metabolic process” (12). For cellular component, the top three were “cell” (14), “cell part” (14), and “organelle” (10). For molecular function, the top three were “binding” (10), “catalytic activity” (10), and “transporter activity” (2) (Figure 3).

**Figure 4:**
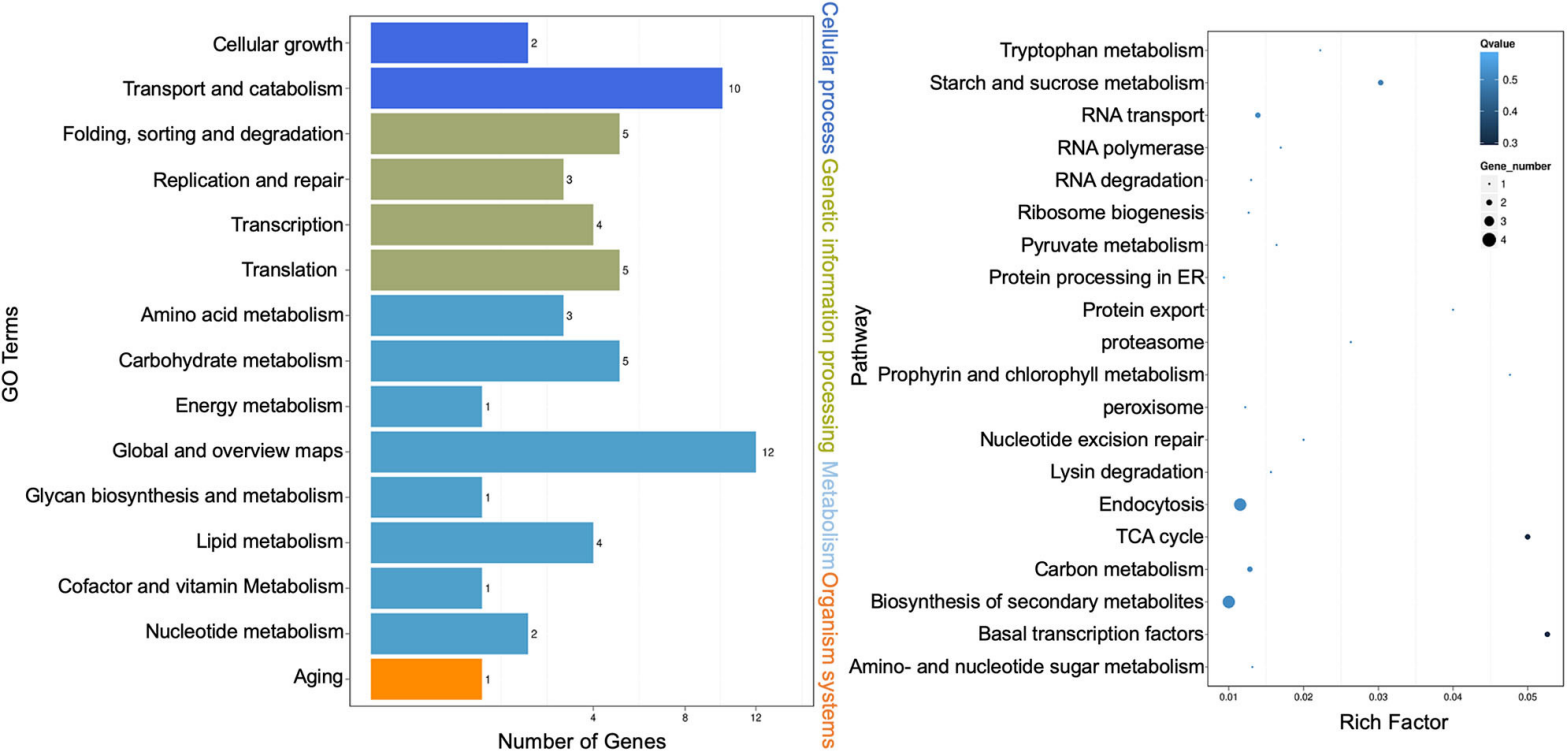
Kyoto Encyclopedia of Genes and Genomes (KEGG) pathway analysis of extracellular microRNA (miRNA) target genes. Bar graph: the left vertical coordinates are 15 KEGG terms of differentially expressed target genes, the bottom horizontal coordinates are the numbers of genes, and the right vertical coordinates indicate the class of Gene Ontology (GO) terms (left panel). Scatter plot: the left vertical coordinates indicate 20 pathways, while the horizontal numbers refer to rich factors (right panel). The rich factor means that the ratio of differentially expressed gene numbers and the number of genes annotated in the pathway. The greater the rich factor, the greater the degree of enrichment. A *Q*-value is the corrected *p*-value ranging from 0 to 1, and a lower value indicates greater pathway enrichment.

Kyoto Encyclopedia of Genes and Genomes (KEGG) pathway enrichment analysis was performed, and both the bar graph and the scatter plot were generated to demonstrate functional classification and pathway assignment of miRNA targets. The top two KEGG pathways were “global and overview maps” (12, metabolism) and “transport and catabolism” (10, cellular process), whereas the “carbohydrate metabolism” (5, metabolism), “folding, sorting, and degradation” (5, genetic information processing), and “translation” (5, genetic information processing) pathways were all ranked in the third place (Figure 4, left panel). A scatter plot of the top 20 pathways indicated that the topmost enriched pathways were “basal transcription factors,” “citric acid cycle (TCA),” and “starch and sucrose metabolism” (Figure 4, right panel). “RNA transport” and “protein export” pathways were also enriched, and more genes were involved in the “endocytosis” and “biosynthesis of secondary metabolites” pathways (Figure 4, right panel). These results are consistent with that Cin1 plays a pleiotropic regulatory function, including membrane transport (Shen et al., 2010).

### Identification of Extracellular Long Noncoding RNA

**Figure 5:**
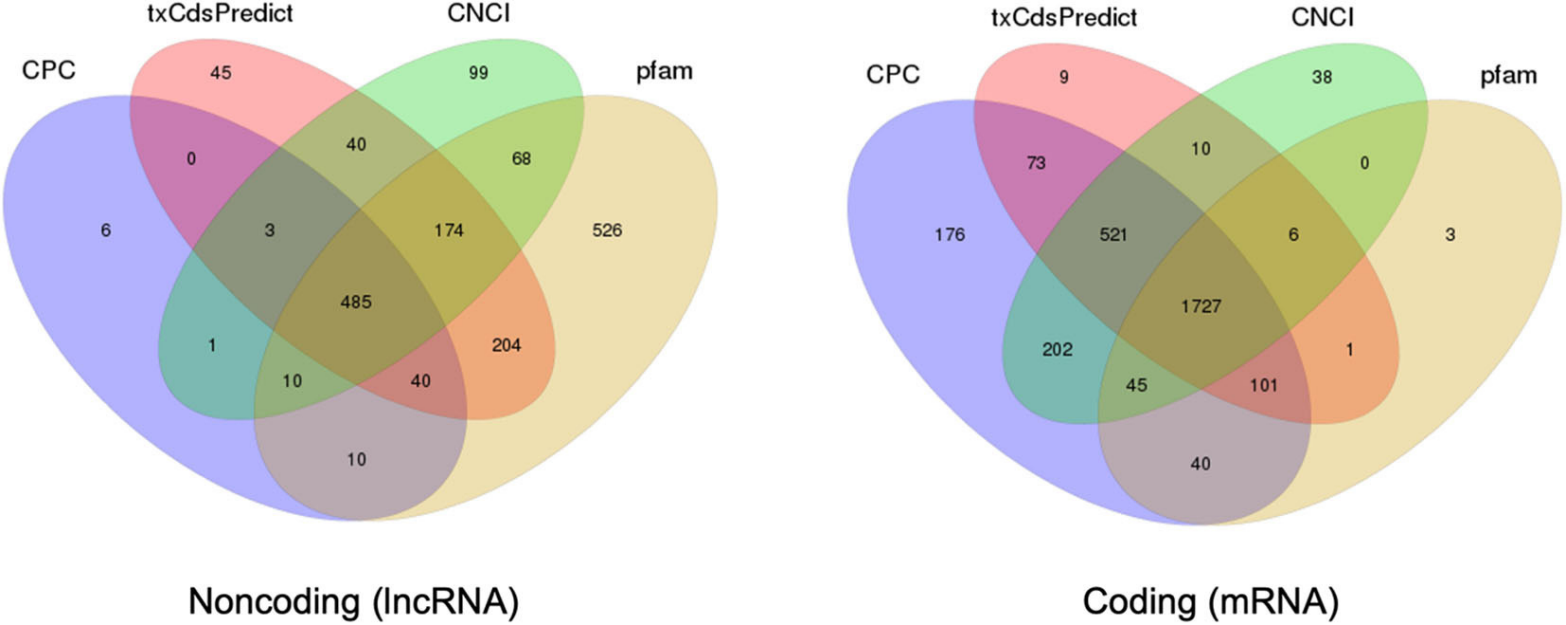
Prediction of long noncoding RNA (lncRNA) and messenger RNA (mRNA) based on the coding ability. Venn diagrams depicting the predication of lncRNA (left diagram) and mRNA (right diagram) by three out of four prediction methods including CPC, txCdsPredict, CNCI, and the Pfam database.

In comparison with extracellular sRNAs sequenced by BGISEQ500, exRNAs of >50 nt were sequenced using an Illumina HiSeq4000 platform by BGI. Approximately 60.5 and 59.7 million clean reads were obtained from WT and Δ*cin1*, with ~61 and ~67% mapping to the *C. deneoformans* genome, respectively. LncRNA and mRNA were distinguished by predicting the coding ability of the transcripts using CPC, txCdsPredict, and CNCI against the Pfam database (Kong et al., 2007; Nawrocki et al., 2009; Sun et al., 2013; Finn et al., 2016; El-Gebali et al., 2019) (Table S7). A separation was made if it satisfies three out of the four prediction methods, as illustrated in the Venn diagrams (Figure 5).

**Figure 6:**
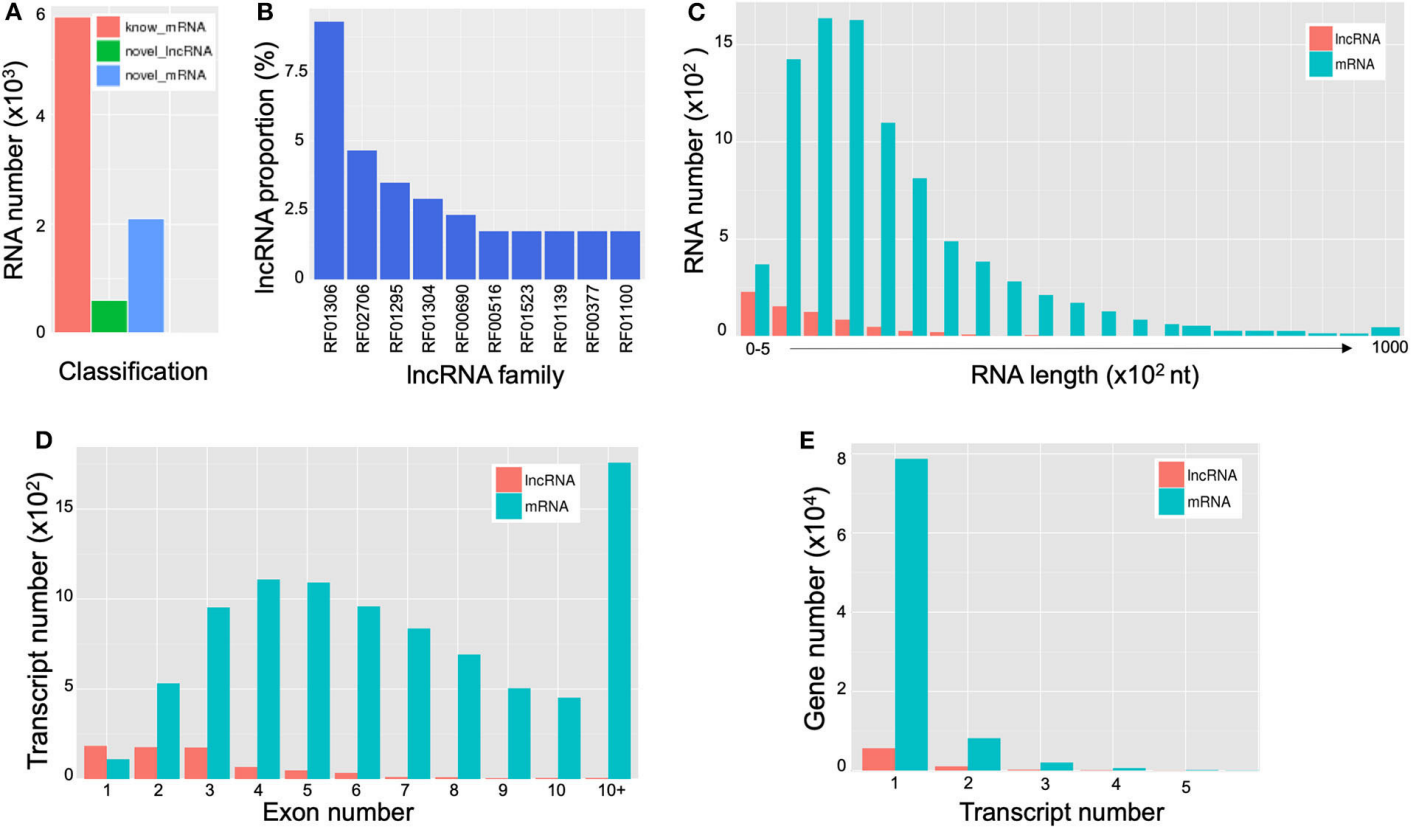
Characterization of extracellular long noncoding RNAs (lncRNAs) and messenger RNAs (mRNAs) of *Cryptococcus deneoformans* wild-type (WT) and Δ*cin1* strains. **(A)** Differential expression; red color represents known mRNA, green color represents lncRNA, and blue color represents novel mRNA. **(B)** Distribution of lncRNA families. The vertical coordinates refer to the percentage of lncRNA, and the horizontal coordinates indicate lncRNA families. **(C)** The RNA length distribution of lncRNA and mRNA. The vertical coordinates refer to RNA numbers, and the horizontal coordinates indicate transcript lengths. **(D)** Distribution of lncRNA and mRNA exon numbers. The vertical coordinates refer to transcript numbers, and the horizontal coordinates indicate exon numbers. **(E)** The distribution of lncRNA and mRNA transcript numbers. The vertical coordinates refer to gene numbers, and the horizontal coordinates refer to transcript numbers.

LncRNAs are RNAs of longer than 200 bp in length; and in contrast to mRNA, they lack the potential for coding proteins. Recently, there has been accumulating evidence that indicates that lncRNA participates in a broad range of cellular processes [reviewed in Meng et al. (2017)]. A total of 690 extracellular lncRNAs were identified from *C. deneoformans* (Table S8). Among them, 572 were from WT and 584 from Δ*cin1*. Also, 600 lncRNAs were found to be differentially expressed using the PossionDis method to detect gene expression (Audic and Claverie, 1997) (Figure 6A). The lncRNAs were also grouped into 10 known lncRNA families when compared against the Rfam dataset using INFERNAL software (Nawrocki et al., 2009; Kalvari et al., 2018) (Figure 6B). Consistent with lncRNA characteristics, cryptococcal lncRNA has a shorter coding sequence (Figure 6C) with fewer exons (Figure 6D), and most contain a single transcript (Figure 6E).

### Functional Annotation of Extracellular Long Noncoding RNA Targets

**Figure 7:**
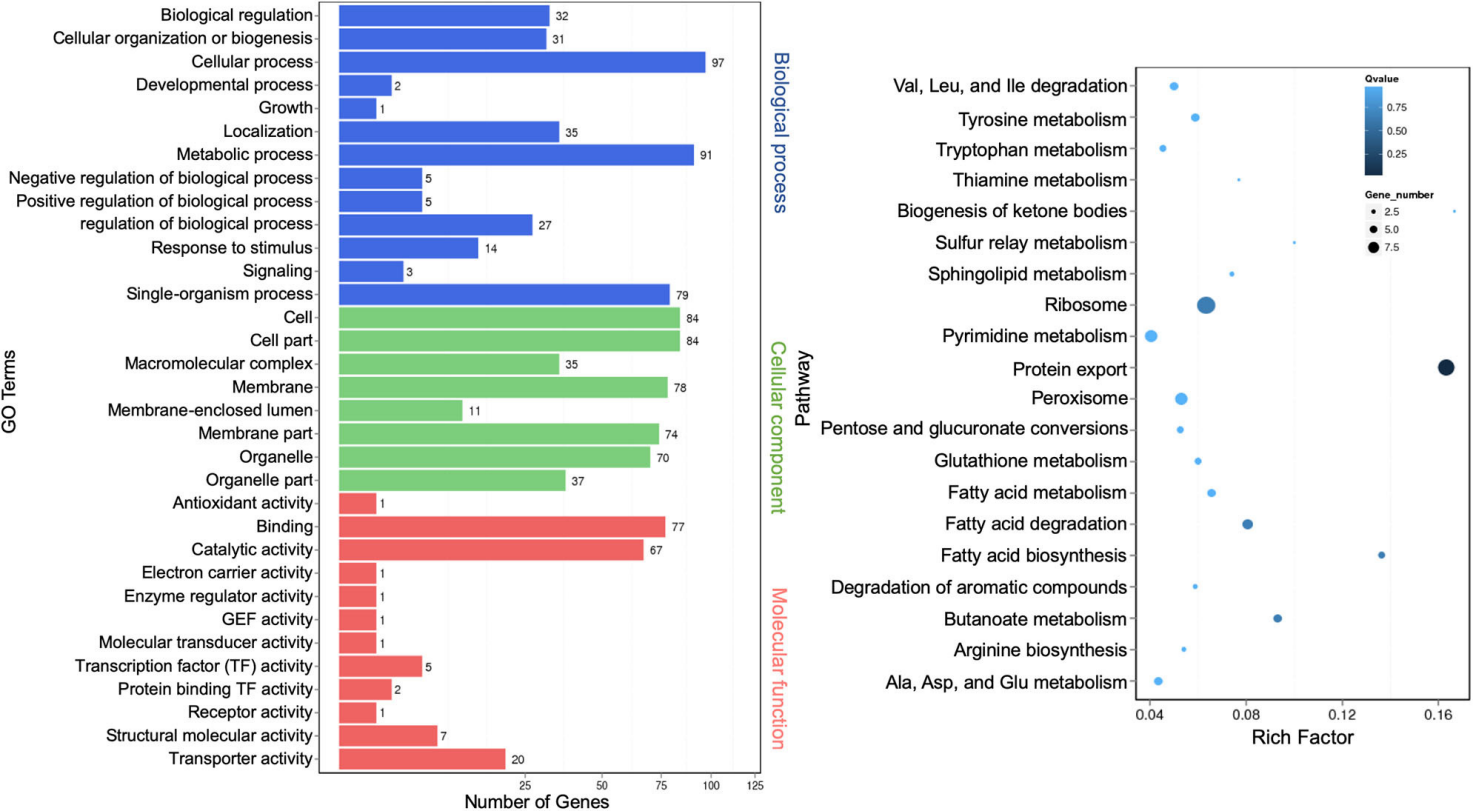
Gene Ontology analysis of differentially expressed extracellular long noncoding RNAs (lncRNA) target genes. A graph plot of Gene Ontology (GO) term enrichment analysis (left panel). The left vertical coordinates refer to 33 GO terms, the bottom horizontal coordinates are the numbers of differentially expressed genes, and the right vertical coordinates are GO classes. A scatter plot of a 20-pathway enrichment analysis (right panel). The left vertical coordinates are pathways, and the horizontal coordinates refer to rich factors.

The regulatory role of lncRNAs in gene expression lies within their functions as either miRNA precursors or miRNA sponge through lncRNA–miRNA interactions (Jarroux et al., 2017; Ulitsky, 2018). Differentially expressed lncRNAs were functionally annotated against GO and KEGG databases. GO terms representing 33 functional categories were identified. The top five categories for biological process are “cellular process” (97), “metabolic process” (91), “single-organism process” (79), “localization” (35), and “biological regulation” (32). The top five categories for cellular components are “cell” (84), “cell part” (84), “membrane” (78), “membrane part” (74), and “organelle” (70). The top three categories for molecular function are “binding” (77), “catalytic activity” (67), and “transporter activity” (20) (Figure 7, left panel). This profile was similar to that of miRNAs (Figure 3). The KEGG pathway analysis showed the top 20 most enriched pathways that were largely similar to miRNA annotation (Figure 4, right panel). Significantly, the scatter plot showed that the topmost enriched pathway involving a relatively high number of genes was “protein export” (Figure 7, right panel).

### Identification and Annotation of Extracellular Messenger RNA

mRNAs are abundant single-stranded RNA molecules directing protein synthesis, and fragmented mRNAs are also proposed to play a role in regulating stability, localization, and translational activity of mRNAs through RNA binding (Batagov and Kurochkin, 2013). In all, 6,899 known extracellular mRNAs were identified (Table S9), with 5,626 and 5,608 mRNAs identified from WT and Δ*cin1*, respectively. In addition, 2,095 novel mRNAs were identified from both strains (Table S10). PossionDis detection for gene expression showed that 5,789 known and 2,096 novel mRNAs were differentially expressed between WT and Δ*cin1* (Figure 6A).

**Figure 8:**
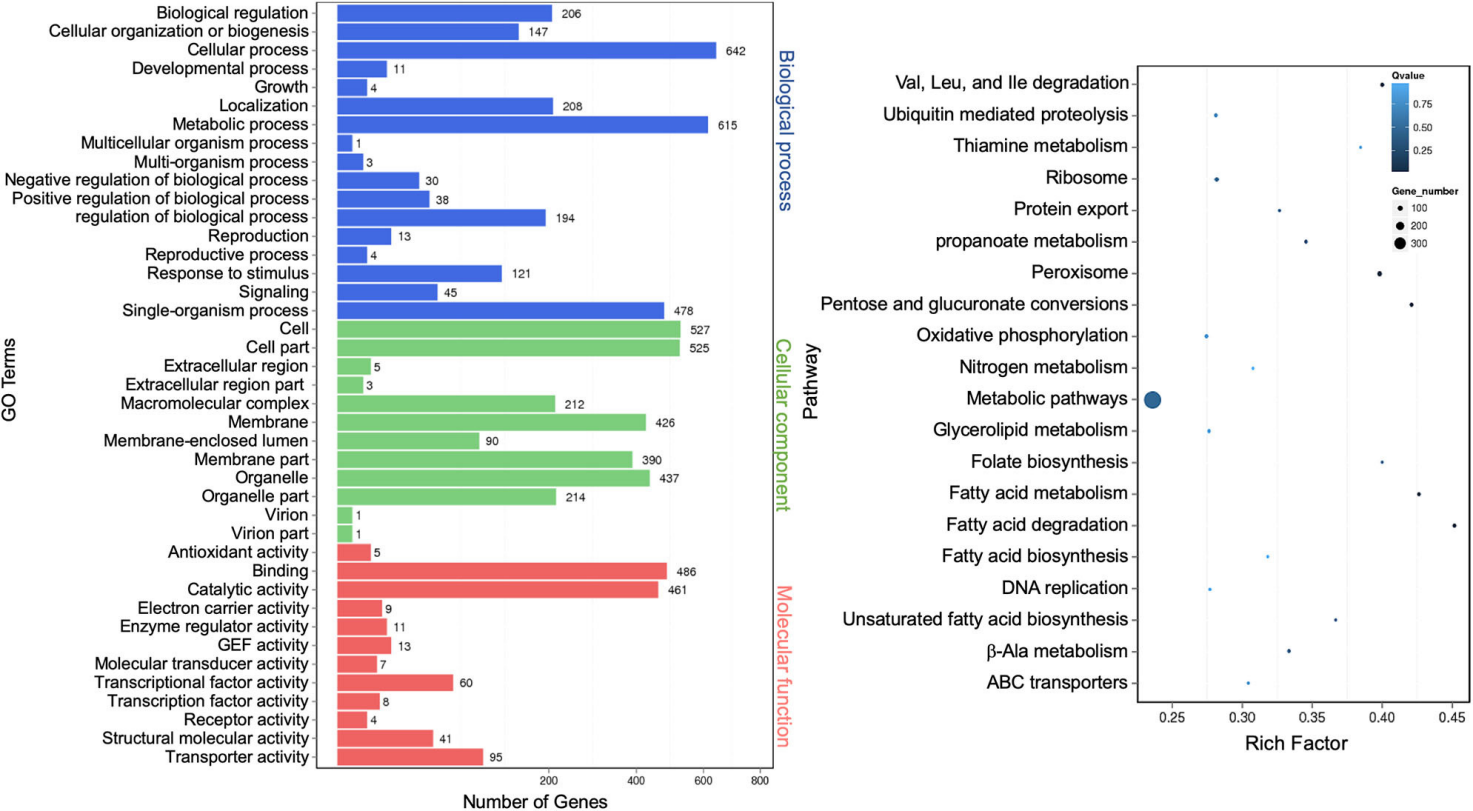
Gene Ontology analysis of differentially expressed extracellular messenger RNAs (mRNAs). A graph plot of Gene Ontology (GO) term enrichment analysis (left panel). The left vertical coordinates refer to 41 GO terms, the bottom horizontal coordinates are the numbers of differentially expressed genes, and the right vertical coordinates are GO classes. A scatter plot of a 20-pathway enrichment analysis (right panel). The left vertical coordinates are pathways, and the horizontal coordinates refer to rich factors.

Differentially expressed known mRNAs were functionally annotated against GO and KEGG databases. GO terms representing 41 functional categories were identified. The top five categories for biological process are “cellular process” (642), “metabolic process” (615), “single-organism process” (478), “localization” (208), and “biological regulation” (206). The top five categories for cellular components are “cell” (527), “cell part” (525), “organelle” (437), “membrane” (426), and “membrane part” (390). The top five categories for molecular function are “binding” (486), “catalytic activity” (461), “transporter activity” (95), “nucleic acid binding transcription factor activity” (60), and “structural molecular activity” (41) (Figure 8, left panel). This profile was similar to that of extracellular miRNAs (Figure 3) and lncRNAs (Figure 7). The result of the top 20 KEGG pathway analysis was also largely similar to that of lncRNA annotation (Figure 7), with the scatter plot showing that the top-ranked enriched pathways were involved in fatty acid, pentose, and glucuronate interconversions, and peroxisome regulation (Figure 8, right panel). “Protein export” remained as one of the most enriched pathways, albeit less than that of the lncRNA targets (Figure 8, right panel).

### Quantitative RT-PCR Validation

**Figure 9:**
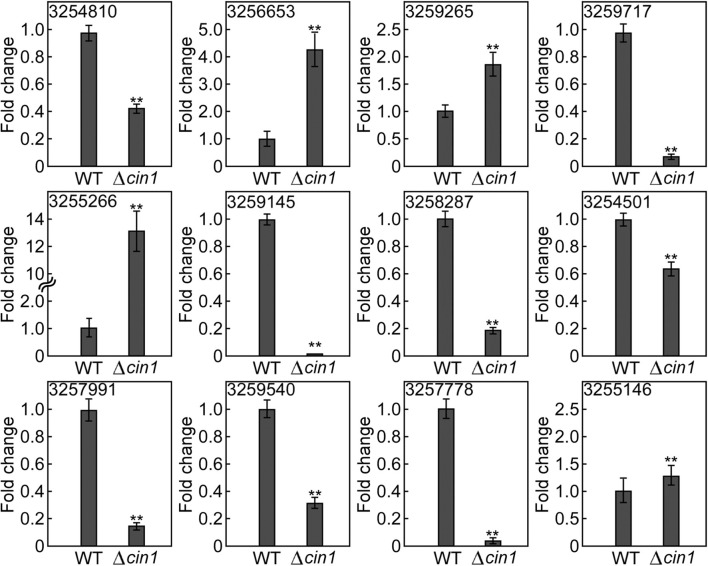
Expression validation by quantitative real-time PCR. The expression of 12 extracellular messenger RNAs (mRNAs) was quantified by qRT-PCR in reference to the expression of the constitutively active *ACT1* gene in *Cryptococcus*. Error bars show standard deviations (*n* = 3), whereas asterisks indicate statistically significant correlations (*p* < 0.05). Gene ID 3254810 (XM_568032.1, a conserved HNG-box protein), 3256653 (XM_569986.1, a BET1-like membrane protein), 3259265 (XM_572299.1, a lipid particle protein), 3255266 (XM_568399.1, a tartrate transporter), 3255266 (XM_568399.1, lysophospholipase), 3254145 (XM_572526.1, a membrane protein), 3254501 (XM_567889.1, alcohol dehydrogenase), 3257991 (XM_571004.1, a hexose transport-related protein), 3257778 (XM_570958.1, a succinate:fumarate antiporter), and 3255146 (XM_568389.1, peptide alpha-*N*-acetyltransferase). 3258287 (XM_571287.1) and 3259540 (XM_573013.1) potentially encode hypothetical proteins.

To validate the accuracy of results from RNA-Seq, we examined the expression of selected mRNAs using quantitative real-time reverse transcription PCR (qRT-PCR). For upregulated mRNAs, we selected XM_568032.1 (3254810, conserved HNG-box protein), XM_569986.1 (3256653, BET1 membrane protein), XM_572299.1 (3259265, lipid particle protein), XM_568399.1 (3255266, tartrate transporter), and XM_568399.1 (3255266, lysophospholipase). For downregulated mRNAs, we chose XM_572526.1 (3254145, a membrane protein), XM_567889.1 (3254501, alcohol dehydrogenase), XM_571004.1 (3257991, hexose transport-related protein), XM_570958.1 (3257778, succinate:fumarate antiporter), and XM_568389.1 (3255146, peptide alpha-*N*-acetyltransferase). These 10 mRNAs exhibited the most apparent differential expression, both up and down. We also selected two mRNAs for hypothetical proteins (XM_571287.1, #3258287 and XM_573013.1, #3259540). qRT-PCR revealed that nine had a similar expression profile (three up and six down) whereas three did not (#3254810, HMG-box protein; #3259717, tartrate transporter; and #3255146, peptide alpha-acetyltransferase) (Figure 9 and Table S1). The reasons for inconsistency remain unknown but could be attributed to variations in sample processing (usage of total cellular RNA instead of vesicular RNA) or errors. While most of these proteins remain functionally uncharacterized, their predicted identities of being membrane and lipid proteins, as well as transport-related proteins, suggest that they are likely involved in intracellular transport. Thereby, their altered expression, either up or down, owing to *CIN1* gene disruption is in accordance with that Cin1 playing an important role in intracellular trafficking.

## Discussion

*Cryptococcus* spp. are unique pathogenic fungi that are characterized by their propensity for the human central nervous system, causing fungal meningitis. Previous studies have provided strong evidence that *Cryptococcus* produces vesicles containing GXM, a capsule precursor, and melanin pigment, and that it secretes laccase, metalloprotein urease, and phospholipases involved in melanin synthesis, stress resistance, and others (Cox et al., 2000, 2001; Olszewski et al., 2004; Rodrigues et al., 2007; Nosanchuk et al., 2008; Vu et al., 2014). Additional studies showed that attenuated expression of genes encoding vesicular proteins, including the small GTPase Sec4/Sav1 protein, the exocyst complex component protein Sec6, and the phospholipid transfer protein Sec14, individually or collectively, affected the secretion of GXM, laccases, ureases, and phospholipases and, thereby, virulence (Yoneda and Doering, 2006; Panepinto et al., 2009; Chayakulkeeree et al., 2011). The composition and architecture of cryptococcal cell walls have also been suggested as the critical factors for the anchoring of melanin pigments, and structural investigation using high-resolution techniques such as solid-state NMR spectroscopy could enhance the understanding of pathogenesis mechanisms (Kang et al., 2018; Chrissian et al., 2020).

Intracellular transport is a complex and highly organized process involving concordant functions of many proteins, in particular, the endocytic adaptor proteins [reviewed in Wang and Shen (2011)]. In contrast to most Sec proteins that are either small GTPases determining transport specificity or vesicle constituents, endocytic proteins function as chaperones interconnecting, and modulating many steps of transport: endocytosis, exocytosis, actin cytoskeleton dynamics, and signal transduction (Bourne et al., 1990; Jenna et al., 2002; Huang and Cai, 2007). The cryptococcal Cin1 protein contains multiple domains, including one Eps15-containing domain, a coiled-coil region, an actin monomer-binding WH2 domain, two SH3 motifs, and a RhoGEF-PH domain (Shen et al., 2010). Cin1 has pleiotropic functions in growth, transport, and the production of virulence factors. Similar to ITSN1, Cin1 functions upstream of the human WASp homolog Wsp1 and Cdc42 GTPase to regulate actin polymerization and organization (Shen et al., 2011, 2012). Collectively, our previous published findings support that the Cin1-Wsp1-Cdc42 regulatory pathway, not described from any other pathogenic fungi, may also contribute to the unique pathogenesis mechanism of *Cryptococcus*.

sRNAs, including miRNAs, siRNAs, and PIWI-interacting RNAs (piRNAs) play a critical role in the regulation of cellular growth and development, as they modulate the expression of target genes via RNA cleavage or transcriptional silencing (Hwang and Mendell, 2006; Osada and Takahashi, 2007). MiRNAs regulate gene expression by targeting the 3′-untranslated region (UTR) of their target mRNAs, whereas siRNAs interfere with gene expression by complementary base pairing to trigger mRNA degradation [reviewed in Bartel (2004), Fabian et al. (2010)]. In a proof-of-principle study, Jiang et al. identified two miRNAs, miR1, and miR2, from a collection of 200 cellular sRNAs of *Cryptococcus deneoformans*. miR1 and miR2 were found to interfere with the expression of *URA5* and *CLC1* genes when inserted in the 3′-UTRs, respectively (Jiang et al., 2012).

In comparison with sRNAs, lncRNAs are RNAs longer than 200 bp in length but are noncoding. There is emerging evidence suggesting that lncRNA also has a broad range of regulatory functions, either as a miRNA precursor or a target/sponge (Paraskevopoulou and Hatzigeorgiou, 2016). However, the existing knowledge regarding lncRNA function is still far less than complete than that for miRNAs. mRNAs are a large family of coding molecules specifying protein sequence information in eukaryotic cells. Previous studies have found that EVs contain a substantial number of mRNAs from their parent cells (Wei et al., 2017). These mRNA molecules are protected from RNase degradation, and those polyadenylated may be capable of encoding polypeptides (Valadi et al., 2007; Lai et al., 2015). In addition, fragmented mRNA resided within EVs could potentially regulate protein functions (Batagov and Kurochkin, 2013).

ExRNAs were previously identified in several pathogenic and non-pathogenic fungi. Peres da Silva et al. identified 344, 423, 145, and 532 miRNAs from *Cryptococcus neoformans* (var. *grubii*), *Candida albicans, Paracoccidioides brasiliensis*, and *Saccharomyces cerevisiae*, respectively (Peres da Silva et al., 2015). The identification of multiple miRNAs, lncRNAs, and mRNAs hypothesized to be involved in vesicle-mediated transport and metabolic pathways led da Salvia et al. to propose that RNA-containing vesicles may be a key determinant for various biological processes, including cell–cell communication and pathogenesis (Peres da Silva et al., 2015). A study by Bielska et al. showed that EVs derived from *Cryptococcus gattii*, a different but related species, could mediate virulence transfer between strains and the characteristics depending on both proteins and RNAs (Bielska et al., 2018). More recently, an RNA sequencing study has also described abundant exRNAs produced by two strains of the mucoralean fungus *Rhizopus delemar* (Bruni et al., 2019). In agreement with these findings, we here identified significant amounts of extracellular miRNAs and siRNAs from *C. deneoformans*. We have also identified a significant amount of extracellular lncRNAs and mRNAs produced by *C. deneoformans*.

Finally, Cin1 was previously characterized to play a pleiotropic function required for the growth, transport, and the production of virulence factors of the fungus (Shen et al., 2010). The Δ*cin1* mutant strain does not produce melanin or capsule, and it also exhibits defects in cytokinesis and growth (Shen et al., 2010). Additional studies suggested that Cin1 functions through a unique Cin1-Wsp1 (Wiskott–Aldrich syndrome protein homolog)-Cdc42 endocytic pathway to regulate growth and virulence, as well as actin dynamics and transport (Shen et al., 2011, 2012). The findings of our current studies are consistent with this conclusion. Analysis of differentially expressed gene targets of miRNA and lncRNA and mRNAs all indicated that Cin1 plays a wide array of functions in cellular processes, composition, and function. All three types of RNAs have roles in RNA and protein export and endocytosis. Further detailed analysis of differential expression of each class of RNAs and their pathways will be required to gain further insight into the regulatory function of Cin1.

## Materials and Methods

### Extracellular Vesicle Isolation

*Cryptococcus deneoformans* (previously *Cryptococcus neoformans* var. *neoformans*) WT JEC21 and Δ*cin1* mutant strains were grown in YPD broth at 30°C for 3 days with 225-rpm rotation (Shen et al., 2010). Yeast cells were precipitated by centrifugation at 4,000 × g for 15 min, and supernatants were recovered. Smaller debris or particles were removed by second centrifugation at 15,000 × g for 15 min at 4°C. The supernatant was then filtered through an ultrafiltration filter with a molecular weight cutoff of 100 kDa (Amicon), and the remaining liquid was precipitated by ultracentrifugation at 100,000 × g for 1 h at 4°C. The precipitated fraction containing membrane fractions was re-suspended in phosphate-buffered saline (PBS), washed twice with PBS, and lyophilized prior to RNA extraction. Owing to the reduced growth of Δ*cin1* in comparison with WT, a larger volume (2×) of YPD was used for its growth. Cultures were grown in duplicated flasks (2×), and vesicle preparations were pooled.

### Extracellular RNA Extraction, Library Construction, and Sequencing

Briefly, vesicular RNAs were extracted with TRIzol (Sigma-Aldrich) and separated by polyacrylamide gel electrophoresis (PAGE), and sRNA bands of ~18–30 nt in size were recovered. 5′ and 3′ adaptors were then added to sRNA prior to cDNA synthesis. The resultant products were purified and amplified by PCR. The PCR yield was quantified and subjected to single-strand circularization (ssDNA circle) for final library construction. According to the BGI protocol, DNA nanoballs (DNBs) were generated with the ssDNA circle by rolling circle replication (RCR) to intensify the fluorescent signals during the sequencing process. The DNBs were then loaded into the patterned nanoarrays, and paired-end reads of 100 bp were read through on a DNBseq™ platform (BGISEQ-500, BGI).

For lncRNA and mRNA characterization, extracted exRNA was first mixed with a biotin-labeled specific probe (Ribo-ZeroTM rRNA Removal Kit) to remove ribosomal RNA (rRNA) and then fragmented. cDNA first strand was synthesized using a TruSeq® Stranded kit (Illumina) and second strand with DNA polymerase I and RNaseH. Double-stranded cDNA was then ligated with an “A” base and a linker and amplified, and the cDNA library generated the following purification. Sequencing was carried out in an Illumina HiSeq4000 platform (BGI). Description and comparison of BGISEQ-500 and Illumina HiSeq for RNA-Seq were previously described by (Zhu et al., 2018).

### MicroRNA Identification, Differential Expression, and Target Prediction

For small exRNA, the sequence tags were subjected to data cleaning analysis to remove transfer RNA (tRNA), rRNA, and other impurities. The clean tags (reads) were then mapped to the *C. deneoformans* genome. Known miRNAs were identified by searching against the miRbase and Rfam reference sRNA database using AASRA software, and novel miRNAs were predicted if they were mapped to the intergenic regions, introns, the reverse repeat sequence of a coding sequence, but not to any other RNAs (Nawrocki et al., 2009; Kozomara and Griffiths-Jones, 2014; Chong et al., 2017; Kalvari et al., 2018). The expression levels of sRNA were calculated using TPM (transcripts per kilobase million; 't Hoen et al., 2008), and differentially expressed sRNAs were screened using ExpDiff (Yang et al., 2017). The false discovery rate (FDR) control method was used to determine the threshold of *p*-value, and the ratio of TPM was used to calculate the fold change in expression. An FDR of < 0.001 and an absolute value of log2-ratio ≥ 1 was set as the threshold for determining the significance of gene expression difference (Kim and van de Wiel, 2008). Once miRNA results were obtained, their target prediction was performed using TargetFinder (Fahlgren and Carrington, 2010; Kielbasa et al., 2010).

### Long Noncoding RNA and Messenger RNA Identification, Coding Ability Prediction, and Differential Expression

The large exRNA clean reads were compared with the *C. deneoformans* genome at National Center for Biotechnology Information (NCBI) using HISAT (Kim et al., 2015) and assembled with StringTie (Pertea et al., 2015). All of the transcript sequences were compared with known lncRNA and mRNA with Cuffcompare (Trapnell et al., 2010). LncRNA and mRNA were distinguished by predicting the coding ability of the transcripts using CPC, txCdsPredict, and CNCI and against the Pfam database (Kong et al., 2007; Nawrocki et al., 2009; Sun et al., 2013; Finn et al., 2016).

### Gene Ontology Term and Kyoto Encyclopedia of Genes and Genomes Pathway Analysis

The differentially expressed miRNA and lncRNA target genes were subjected to GO term and KEGG pathway enrichment analysis. GO provides a common descriptive framework and functional annotation and classification for analyzing gene set data (www.geneontology.org; Ashburner et al., 2000; The Gene Ontology Consortium, 2019), whereas the KEGG pathway database is a recognized and comprehensive database including all known biochemical pathways (www.annotation.jp/KEGG; Kanehisa and Goto, 2000; Kanehisa et al., 2016). GO term and KEGG pathway analyses for mRNA genes were the same as for miRNA and lncRNA target genes.

### Quantitative RT-PCR Verification

As a verification step for RNA sequencing, selected mRNAs were examined for expression by qRT-PCR. One microgram of total RNA was reverse transcribed into first-strand cDNA using the oligo(dT) or random primers and M-MLV Reverse Transcriptase (Invitrogen) following previously described methods (Chong et al., 2017; Liu et al., 2018). The expression of the constitutively expressed actin gene *ACT1* was used as an internal control. Primers used for qRT-PCR are listed in Table S2. qRT-PCR was performed with an ABI 7500 Fast Real-Time System, and transcripts were analyzed by 7500 System SDS software. To compare the relative abundance of target gene transcripts in different samples, the average threshold cycle (Ct) was normalized to *ACT1* for each sample as 2^−Δ*Ct*^ [–ΔCt = (Ct, target gene-Ct, *ACT1*)]. Fold changes between different samples were calculated as 2^−ΔΔ*Ct*^ [–ΔΔCt = (Ct, experimental-Ct, *ACT1*) – (Ct, control-Ct, *ACT1*)].

## Data Availability Statement

This data can be found here: https://www.ncbi.nlm.nih.gov; BioSample accessions SAMN14775367, SAMN14775368, SAMN14775369.

## Author Contributions

All authors listed have made substantial, direct, and intellectual contribution to the work, and approved it for publication.

## Conflict of Interest

The authors declare that the research was conducted in the absence of any commercial or financial relationships that could be construed as a potential conflict of interest.

## Supplementary Material

Table S1 Differential expression of secreted mRNA between *C. deneoformans* JEC21 and *cin1* strains revealed by RNA-Seq.

Table S2 Primers used in this study.

Table S3 miRNAs identified from *C. denewformans* JEC21 strain.

Table S4 miRNAs identified from *C. denewformans cin1* mutant strain.

Table S5 Novel miRNAs identified from *C. denewformans*.

Table S6 Novel siRNAs identified from *C. denewformans*.

Table S7 Coding and noncoding RNAs identified from *C. denewformans*.

Table S8 Novel lncRNAs identified from *C. denewformans*.

Table S9 Known mRNAs identified from *C. denewformans*.

Table S10 Novel mRNAs identified from *C. denewformans*.
