# Supplementary figures and images for: Transcriptomic Analysis of Extracellular RNA Governed by the Endocytic Adaptor Protein Cin1 of Cryptococcus deneoformans
Source: Front Cell Infect Microbiol. 2020 Jun 23;10:256. doi: 10.3389/fcimb.2020.00256 (PMC7324655; doi:10.3389/fcimb.2020.00256)

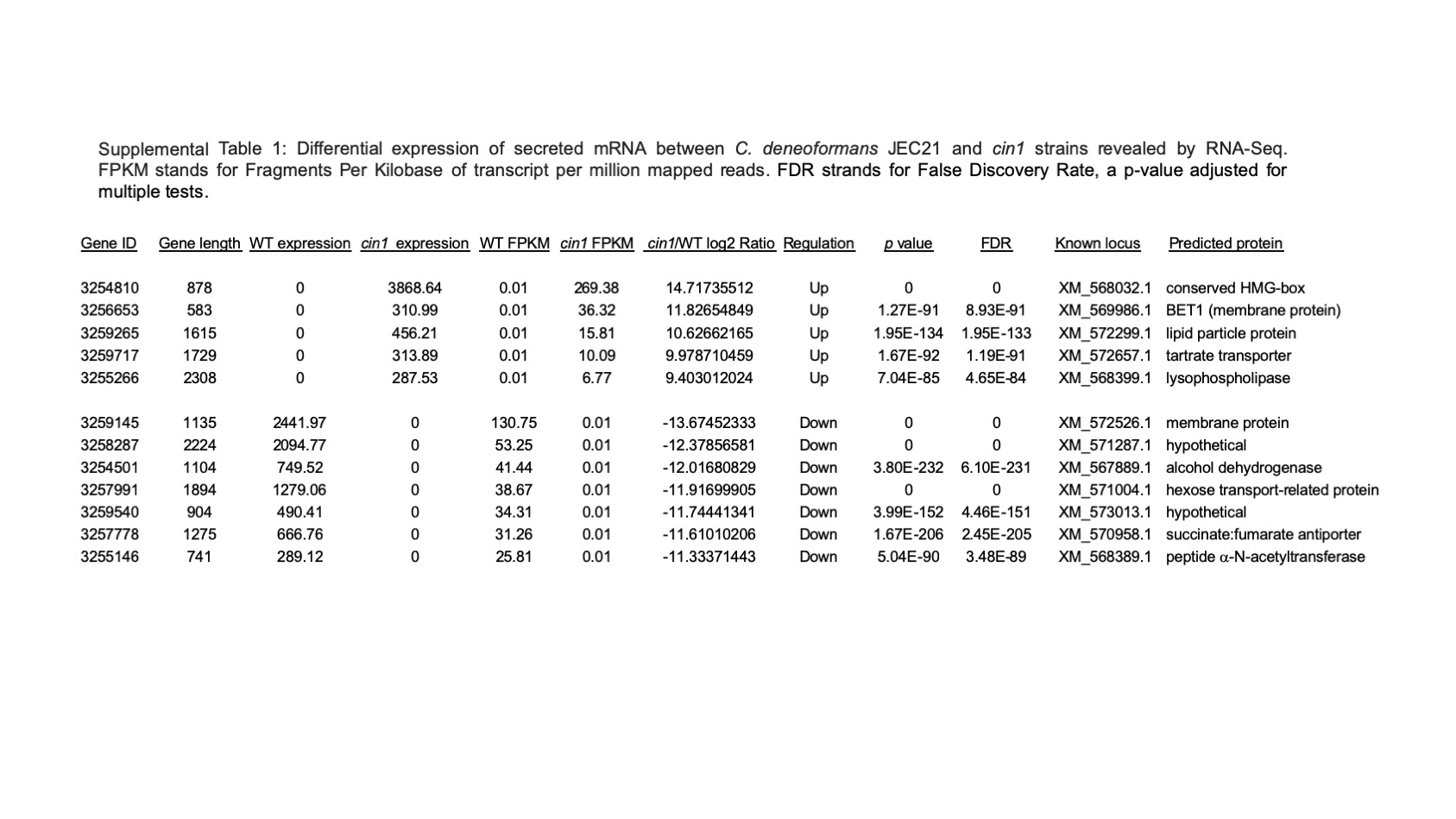

Supplement: Table S1 — Differential expression of secreted mRNA between C. deneoformans JEC21 and cin1 strains revealed by RNA-Seq. [file Data_Sheet_1.zip › Table S1.jpeg]

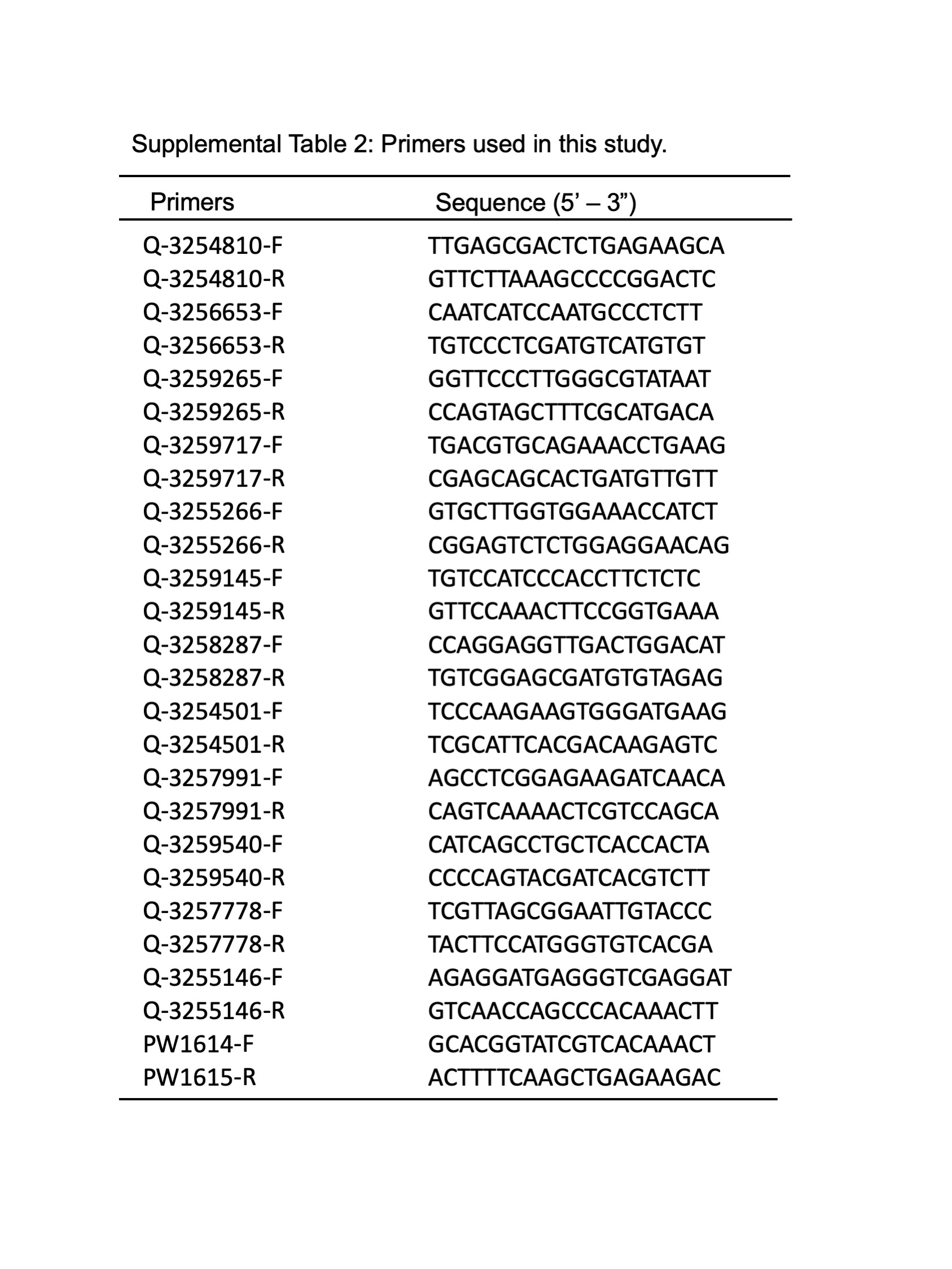

Supplement: Table S1 — Differential expression of secreted mRNA between C. deneoformans JEC21 and cin1 strains revealed by RNA-Seq. [file Data_Sheet_1.zip › Table S2.jpeg]
